# 

**Published:** 1822-07

**Authors:** 


### TO CORRESPONDENTS.

*The Editor is desirous of stating, that, in consequence of an accumulation of engagements, he has been obliged to avail himself of the services of a professional Gentleman, by whom the* Half-yearly Retrospect, *and the* Review of Mr. CLARKE's Work on the Diseases of Females, *inserted in the present Number, have been written.*

*The* Retrospect *having precluded the* Original Department, *as well as that devoted to the* Monthly Bibliography, *the Editor hopes to be able to insert a great portion of the Communications and Reviews on hand in the next Number of the Journal.*

